# RTK25: A Comprehensive Molecular Profiling Strategy in Cholangiocarcinoma Using an Integrated Bioinformatics Approach

**DOI:** 10.3390/ph14090898

**Published:** 2021-09-03

**Authors:** Brinda Balasubramanian, Simran Venkatraman, Tavan Janvilisri, Tuangporn Suthiphongchai, Siriporn Jitkaew, Jittiyawadee Sripa, Rutaiwan Tohtong

**Affiliations:** 1 Graduate Program in Molecular Medicine, Faculty of Science, Mahidol University, Bangkok 10400, Thailand; balasubramanian.bri@student.mahidol.edu (B.B.); simran.ven@student.mahidol.ac.th (S.V.); 2 Department of Biochemistry, Faculty of Science, Mahidol University, Bangkok 10400, Thailand; tavan.jan@mahidol.ac.th (T.J.); tuangporn.sut@mahidol.ac.th (T.S.); 3 Age-Related Inflammation and Degeneration Research Unit, Department of Clinical Chemistry, Faculty of Allied Health Sciences, Chulalongkorn University, Bangkok 10330, Thailand; Siriporn.ji@chula.ac.th; 4 College of Medicine and Public Health, Ubon Ratchathani University, Ubon Ratchathani 34190, Thailand; jittiyawadee.s@ubu.ac.th

**Keywords:** cholangiocarcinoma, targeted therapy, biomarkers, receptor tyrosine kinases, precision medicine

## Abstract

Cholangiocarcinoma (CCA) is a heterogeneous group of malignancies that primarily originate from the bile duct. Tumor heterogeneity is a prime characteristic of CCA and considering the scarcity of approved targeted therapy drugs, this makes precision oncology impractical in CCA. Stratifying patients based on their molecular signature and biomarker-guided therapy may offer a conducive solution. Receptors tyrosine kinases (RTK) are potential targets for novel therapeutic strategies in CCA as RTK signaling is dysregulated in CCA. This study aims to identify targetable RTK profile in CCA using a bioinformatic approach. We discovered that CCA samples could be grouped into molecular subtypes based on the gene expression profile of selected RTKs (RTK25). Using the RTK25 gene list, we discovered five distinct molecular subtypes of CCA in this cohort. Tyrosine kinase inhibitors that target each RTK profile and their subsequent molecular signatures were also discovered. These results suggest that certain RTKs correlate with each other, indicating that tailored dual inhibition of RTKs may be more favorable than monotherapy. The results from this study can direct future investigative attention towards validating this concept in in vivo and in vitro systems. Ultimately, this will facilitate biomarker-guided clinical trials for the successful approval of novel therapeutic options in CCA.

## 1. Introduction

Cholangiocarcinoma (CCA) is a group of heterogeneous malignancies which originate in the biliary tract, the duct that carries bile to the liver. CCA is generally asymptomatic and aggressive in nature, which makes its diagnosis and treatment problematic. CCA, although rare, is a cause for global concern due to its increasing incidence and high mortality rate. There is a deficit of early detection techniques and efficacious treatment options, which result in a dismal prognosis, with a less than 10% five-year survival rate [1,2]. Currently, the treatment options are limited based on disease progression. Surgical intervention is the only treatment with curative intent for patients in early stages of CCA [3]. Moreover, a combination of cytoreductive surgery (CRS) and hyperthermic intraperitoneal chemotherapy (HIPEC) is performed to improve overall survival (OS) in patients with advanced metastatic CCA [4,5]. Furthermore, patients with unresectable CCA are limited to palliative chemotherapy [1]. However, the cancer recurrence rate in patients undergoing surgery is high and the patients in the most advanced stage are unable to tolerate the aggressiveness of systemic therapy [6,7]. In addition, the highly heterogeneous nature of CCA impede the effectiveness of available treatment in CCA. As the disparities at genomic, epigenetic, and molecular levels contribute to the tumor heterogeneity, a more stratified approach is required to molecularly profile CCA patients to better prescribe therapeutics. There is increasing evidence that support the use of guided targeted therapy drugs by accurately treating patients according to their distinct molecular profiles [7]. The advent of molecular profiling studies identified CCA subtypes with *IDH* mutants and *FGFR2* fusions. Currently, inhibitors of IDH and FGFR are being evaluated in clinical trials for specific subtypes of patients with *IDH* mutations (NCT02989857, NCT04521686) and *FGFR2* fusions (NCT03656536, NCT03773302, NCT01752920) [8]. The outcomes of clinical trials has encouraged the approval of Pemigatinib, an FGFR2 inhibitor, for previously treated patients with advanced metastatic CCA harboring FGFR2 fusions [9]. The success of this trial encourages more investigations to find other key driving receptors that might be actionable targets in treating CCA. Hence, targeted therapy has emerged recently as a potential measure against CCA.

Receptor tyrosine kinases (RTKs) have a role in triggering several pathways such as cell proliferation, morphogenesis, survival, invasion, and migration in cancer [10]. Oncogenic activation of RTKs can be initiated by several oncogenic mechanisms such as gain-of-function mutations, overexpression and amplification, chromosomal rearrangements, and autocrine loop (where the cell expressing the target receptors are also the ligand secreting cells). In addition, there are emerging mechanisms such as miRNA, epigenetic modulations that can regulate the activity of RTK signaling in cancer [11]. ALK, EGFR, FGFR, FLT3, NTRK, and VEGFR are the most well-known RTKs with aberrant signaling in different cancer types. This is characterized by the approval of several tyrosine kinase inhibitors (TKIs) targeting these RTKs [12]. Similarly, aberrant RTK signaling is known in CCA. EGFR, ERBB2, and MET expression are documented and are associated with poor prognosis. Moreover, *FGFR2* translocations and ROS1 kinase fusion proteins are also known in CCA. In addition, CCA cells also secrete platelet-derived growth factor B (PDGFB) that activates its associated receptor PDGFR [1,13]. Furthermore, CCA cells also express vascular endothelial growth factor A (VEGFA) which is correlated with increased vascular density which in turn correlates with cancer progression, metastasis, and prognosis in intrahepatic and hilar cholangiocarcinoma [14].

Altogether, this implicates RTKs as potential therapeutic targets in CCA management. RTK inhibitors, a class of targeted therapeutics that target the active site of RTKs to prevent their phosphorylation and subsequent activation of downstream signaling cascades, can be vital for CCA treatment [12]. Several other kinase inhibitors that target RTKs are currently in various phases of clinical trials in CCA. However, despite numerous clinical trials on RTK inhibitors, there is still a scarcity of targeted therapy drugs in CCA. We previously reported the importance of patient stratification for successful clinical trials which would eventually result in increased approved targeted therapy in CCA [15]. Recognizing that many of these drugs will be off patent in the coming years [16], their biosimilars may make targeted therapy and precision medicine more affordable to patients. Nevertheless, the molecular signature of RTKs and their role in CCA pathogenesis is still poorly understood. Our study aims to identify distinct RTK profiles to help understand their significance in CCA. The findings from this study will provide necessary insights about the landscape of RTKs in CCA to discern TKIs and novel combinations that target these profiles. Moreover, our study identifies specific areas with knowledge gaps in CCA biology and treatment and can direct future investigative attention towards precision therapy. Ultimately, this will facilitate biomarker-guided clinical trials and redefine patient stratification for personalized medicine in CCA.

## 2. Results

### 2.1. Ectopic Expression of Certain RTKs Is Identified in CCA

**Figure 1:**
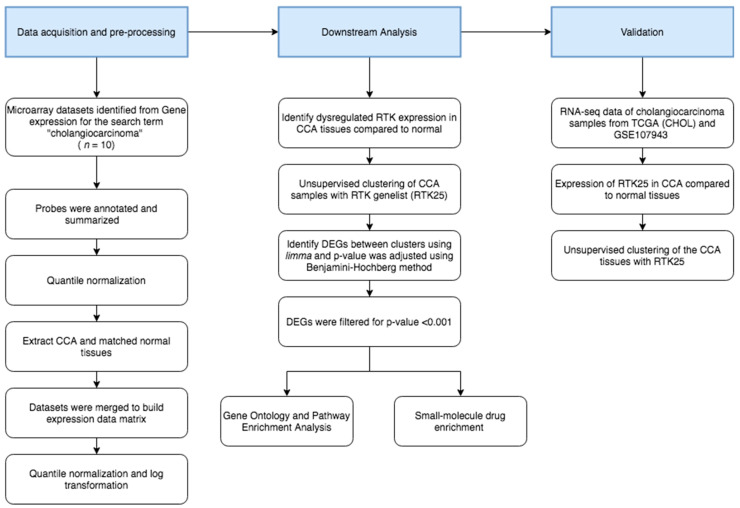
Schematic workflow of the meta-analysis. Microarray gene expression data from multiple independent studies was obtained from the Gene Expression Omnibus (GEO) database. The collated data was pre-processed, and a gene expression matrix was constructed and normalized for further analysis. See materials and methods for details. CCA, cholangiocarcinoma; RTK, receptor tyrosine kinase; DEGs, differentially expressed genes; TCGA, The Cancer Genome Atlas.

**Figure 2:**
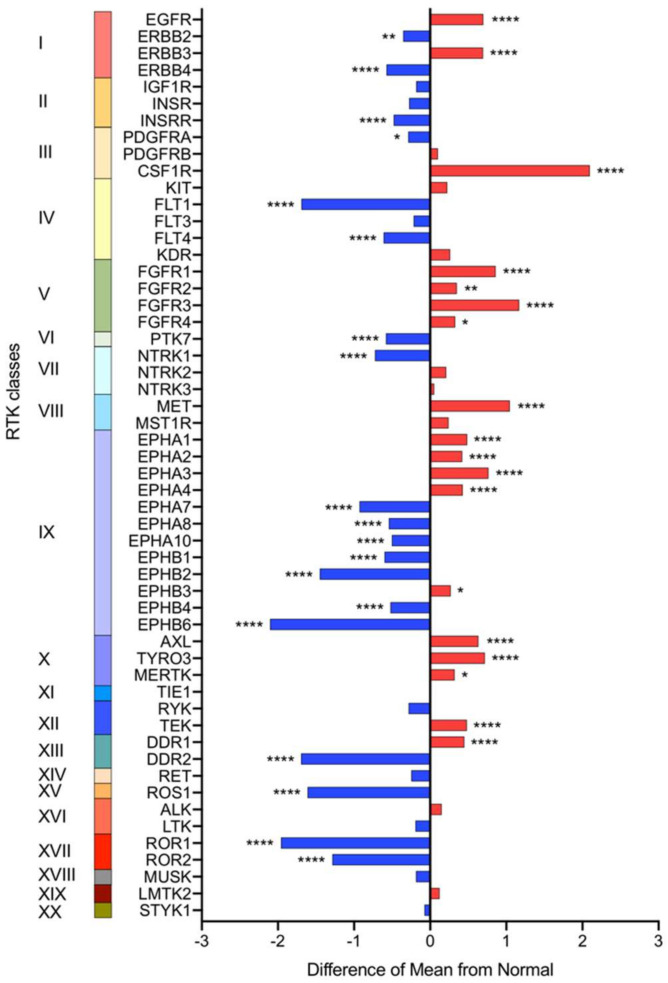
Pairwise comparison of RTKs between CCA and normal tissues. The gene expression of 54 RTKs from 20 different classes were compared between CCA and normal tissues. The difference in mean expression of each RTKs in CCA tumors (*n* = 704) from normal tissues (*n* = 165) were calculated and plotted as a bar graph. The RTKs with higher mean expression in CCA than normal tissues are indicated with red bars whereas the RTKs with lower mean expression are indicated with the blue bars. The method for differential analysis between tumor and normal was one-way ANOVA testing followed by Holm-Sidak post-hoc analysis. * *p* < 0.05, ** *p* < 0.01, **** *p* < 0.0001.

Overexpression or ectopic expression of RTKs can lead to dysregulated signaling in cancer. While it is known from several reports that RTK signaling is frequently dysregulated in CCA, there are not many studies that investigate all the receptors from the RTK family holistically. Hence, to identify the differentially expressed RTKs in CCA tissues, we constructed a gene expression matrix using the 10 independently collated datasets obtained from GEO, consisting of 704 CCA tumors and 165 normal tissues, as listed in Table 1. The schematic workflow of this meta-analysis is illustrated (Figure 1). RTK gene list was obtained from HUGO Gene Nomenclature Committee (HNGC) [17] We performed pairwise comparisons of the mRNA expression of 54 RTKs between CCA and normal tissues, which resulted in a total of 37 significantly differentially expressed RTKs in CCA, the difference of mean was plotted to illustrate the ectopic expression of the RTKs from all subfamilies (Figure 2). *EGFR, ERBB3, CSF1R, FGFR1-4, MET, EPHA1-4, EPHB3, AXL, TYRO3, MERTK, TEK,* and *DDR1* were expressed in significantly higher levels in CCA when compared with normal tissues. However, *ERBB2, ERBB4, INSRR, PDGFRA, FLT1, FLT4, PTK7, NTRK1, EPHA7, EPHA8, EPHA10, EPHB1, EPHB2, EPHB4, EPHB6, DDR2, ROS1, ROR1*, and *ROR2* were expressed in significantly lower levels in CCA compared to the normal tissues. Together, these results suggest that certain RTKs are differentially expressed exclusively in CCA tissues, thereby insinuating that certain RTKs may be actionable targets for treatment with TKIs in CCA.

### 2.2. Molecular Profiling Using RTK25 Identifies Five Instrisic Subtypes with Distinct RTK Expression Profiles in CCA

**Figure 3:**
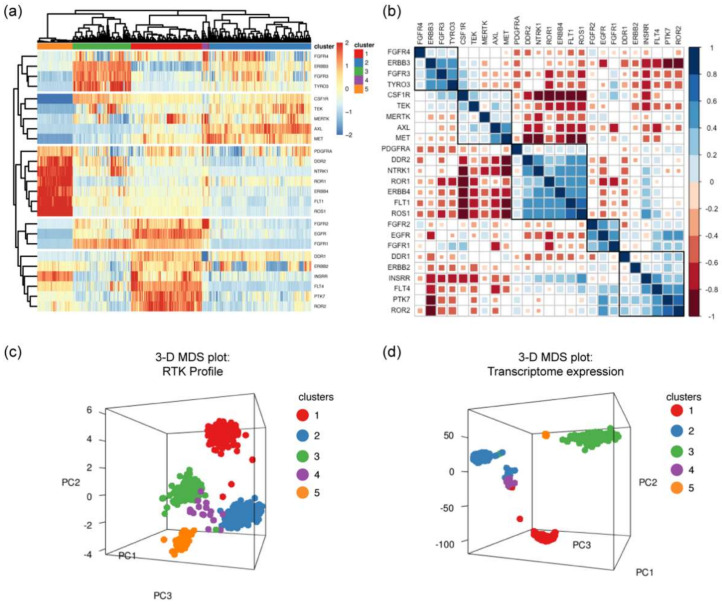
Molecular subtypes with distinct RTK profile can be identified using RTK25. (**a**) Hierarchical clustering was performed on the CCA tissues (*n* = 704) based on the gene expression of selected RTKs (RTK25). The heatmap indicates the expression values (red indicates high expression and blue indicates low expression) of RTK25 in each cluster (red = 1, blue = 2, green = 3, purple = 4, and orange = 5). (**b**) Correlation matrix shows significant (*p* < 0.05) Pearson correlation coefficients. Color indicates correlation (blue, positive correlation (*r* = 0 to 1); red, negative correlation (*r* = −1 to 0)). Area of the square indicates the value of the corresponding Pearson’s correlation coefficients. Multi-dimensional scaling (MDS) plot of 704 CCA samples based on the expression of (**c**) RTK genes (*n* = 25) and (**d**) all the common genes from the merged expression data (*n* = 13454). PC1: first MDS component (*x*-axis); PC2: second MDS component (*y*-axis); PC3: third MDS component (*z*-axis).

Recognizing the significance of certain RTKs expression on CCA tumors, we speculated that due to the heterogeneity of CCA tumors, the samples would comprise of distinct expression profiles of RTKs. We hypothesized that the CCA samples could be separated into intrinsic molecular subtypes based on their RTK gene expression profile. To investigate this hypothesis, we performed unsupervised hierarchical clustering using the significantly differentially expressed RTKs against the 704 CCA samples. Hierarchical clustering analysis employs an algorithm to mathematically group samples based on gene expression data. For the optimal separation of the clusters, our final gene list resulted in 25 RTKs (RTK25) out of the 37 RTKs that were significantly differentially expressed in CCA compared with normal tissues (Table S1). With unsupervised hierarchical clustering analysis, we observed that the 704 CCA samples grouped into 5 different clusters, each with a distinct RTK expression profile (Figure 3a). Since the similarly expressed RTKs were also clustered into groups, we sought to identify the relationship amongst RTKs using the pairwise Pearson correlation coefficient (r) analysis. The results revealed the degree of correlation among the RTK25, positive correlation coefficients (r > 0) indicated co-expressed RTKs whereas negative correlation coefficients (r < 0) indicated antagonistic relationship between RTKs. With the ordered correlation matrix, we observed that positively correlated RTKs were grouped similarly to the clustering analysis (Figure 3b). Based on the unsupervised clustering and pairwise Pearson correlation analysis, we discovered that there are five distinct groups of RTK expression profiles in this cohort, we named these clusters numerically, 1, 2, 3, 4, and 5 (Figure 3c). *DDR1*, *EGFR*, *ERBB2*, *FGFR1*, *FGFR2*, *FLT4*, *INSRR*, *PTK7*, and *ROR2* are highly expressed in the first group, cluster 1. Whereas *AXL*, *MET*, *MERTK*, and *TEK* were highly expressed in cluster 2. Likewise, *ERBB3*, *FGFR3*, and *TYRO3* were highly expressed in cluster 3. Cluster 4, the smallest group, had a high expression of *AXL*, *FGFR2*, *FGFR4*, *MERTK*, and *MET*. Finally, *ERBB4*, *DDR2*, *FLT1*, *NTRK1*, *PDGFRA*, *ROR1*, and *ROS1* were expressed highly in cluster 5. To validate whether these groups could form intrinsic molecular subtypes in this cohort, we performed multidimensional scaling (MDS) analysis using (a) RTK genes (*n* = 25) and (b) all the common genes from the merged expression data (*n* = 13454). MDS analysis is a method to visually represent the similarity or dissimilarity among samples. The first MDS plot (Figure 3c) shows that the CCA samples were grouped into five distinct clusters based on the RTK25 gene expression. The second MDS plot (Figure 3d) confirms that the same CCA samples were separated into five distinct clusters. This analysis shows that there were five molecular subtypes of CCA in this cohort, and these groups could be characterized by their own unique RTK signature. Together, these results illustrate distinct molecular subtypes with targetable RTK profiles in CCA that could be identified using RTK25 gene list.

### 2.3. Differentially Expressed Genes amongst CCA Clusters Show That Each Subtype Has an Unique Molecular Signature

**Figure 4:**
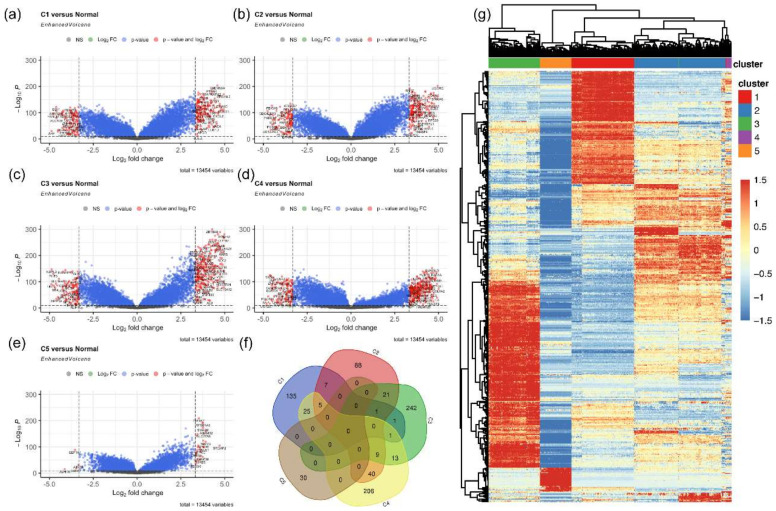
Differentially expressed genes (DEGs) in each cluster compared to normal tissues. Volcano plots comparing the fold change in clusters and normal tissues illustrate DEGs in (**a**) cluster 1, (**b**) cluster 2, (**c**) cluster 3, (**d**) cluster 4 and (**e**) cluster 5. Statistically significant DEGs which meet the fold change cut-off are represented by red dots (adjusted *p* < 0.001), blue dots represent significant DEGs beyond the log2 fold change cut-off (adjusted *p* < 0.001), and grey dots represent non-significant genes. (**f**) Venn diagram of upregulated DEGs that are unique in each cluster. (**g**) Heatmap of the upregulated DEGs that are unique in each cluster.

Having established the five molecular subtypes identified for our CCA cohort, we aimed to survey the transcriptomic signatures that define these tumors against normal tissues. Hence, we performed differential gene expression (DGE) analysis between each of the five clusters and normal tissues using LIMMA statistical model and selected genes with a Benjamini–Hochberg adjusted *p*-value lower than 0.001. This analysis resulted in a total of 108 downregulated and 175 upregulated DEGs in cluster 1, 103 downregulated and 171 upregulated DEGs in cluster 2, 116 downregulated and 288 upregulated in cluster 3, 271 downregulated and 299 upregulated in cluster 4 and finally, 5 downregulated and 30 upregulated in cluster 5 (Figure 4a–e). We noticed that the samples in cluster 5 were most similar to the normal group and resulted in very few DEGs compared to the other clusters. In addition to clustering analysis, we also confirmed that each cluster was independent of each other using DGE analysis of every cluster against other clusters (Figure S1). The resultant upregulated DEGs in each cluster were plotted using a Venn diagram generator, which identified DEGs that are unique to each cluster (135 in cluster 1; 88 in cluster 2; 242 in cluster 3; 206 in cluster 4; 30 in cluster 5) (Figure 4f). The expression of these DEGs, which are exclusively upregulated in each cluster, are shown in the heatmap (Figure 4g). Unsupervised clustering analysis separated the samples into clusters similar to the RTK profile (Figure 3a). Together, these results demonstrate that the groups profiled using RTK25 not only have a distinct RTK expression profile but also have their own unique overall gene expression signature.

### 2.4. Gene Ontology and Pathway Enrichment Analysis

To identify the functional role of the molecular signature of each cluster, we performed pathway enrichment analysis in ‘Enrichr’ using the DEGs unique to each cluster. *p*-value cutoff was set at 0.05 and the significantly enriched pathways were ordered by combined score. The top 10 enriched GO terms and pathways from the BioPlanet, KEGG, MSigDB, and Wikipathways databases for each cluster are illustrated as bubble plots (Figures S2–S6). Major oncogenic signaling pathways, such as ‘VEGF signaling’, ‘angiogenesis’, ‘focal adhesion’, ‘epithelial–mesenchymal transition’, and inflammation associated pathways such as ‘interferon’ and ‘interleukin’ pathways were significantly enriched in cluster 1 (Figure S2A,B). In cluster 2, cellular respiratory and metabolic pathways such as electron transport chain, oxidative phosphorylation, and mitochondrion pathways were significantly enriched. Additionally, the ‘p53 pathway’ was also enriched in this cluster (Figure S3A,B). In cluster 3, intracellular signaling pathways such as ‘Wnt’ and ‘KRAS’ signaling and ‘Hedgehog’ signaling were significantly enriched. Moreover, metabolic pathways such as ‘lipid metabolism’, ‘bile acid metabolism’, and ‘fatty acid metabolism’ and cellular respiratory pathways such as ‘TCA cycle’, ‘disorder of the Krebs cycle’, and ‘oxidative phosphorylation’ were also enriched (Figure S4A,B). Inflammation-associated pathways such as ‘interferon’ and ‘interleukin’ pathways were enriched in cluster 4, predominantly. Moreover, metastasis-related pathways such as ‘cell adhesion’, ‘ECM-receptor interaction’, ‘epithelial–mesenchymal transition’, ‘focal adhesion’, and ‘cadherin binding’ were also enriched in cluster 4 (Figure S5A,B). Finally, intracellular signal transduction pathways such as ‘p38 MAPK’, ‘ERK’, ‘BMP’, ‘TGF-beta’ signaling, and ‘CREB’ phosphorylation were enriched in cluster 5 (Figure S6A,B). Altogether, this data shows that RTKs had diverse roles in CCA therefore, they could serve as actionable targets for therapeutic intervention in CCA patients.

### 2.5. Validation of RTK25 Gene List for Molecular Profiling in CCA

**Figure 5:**
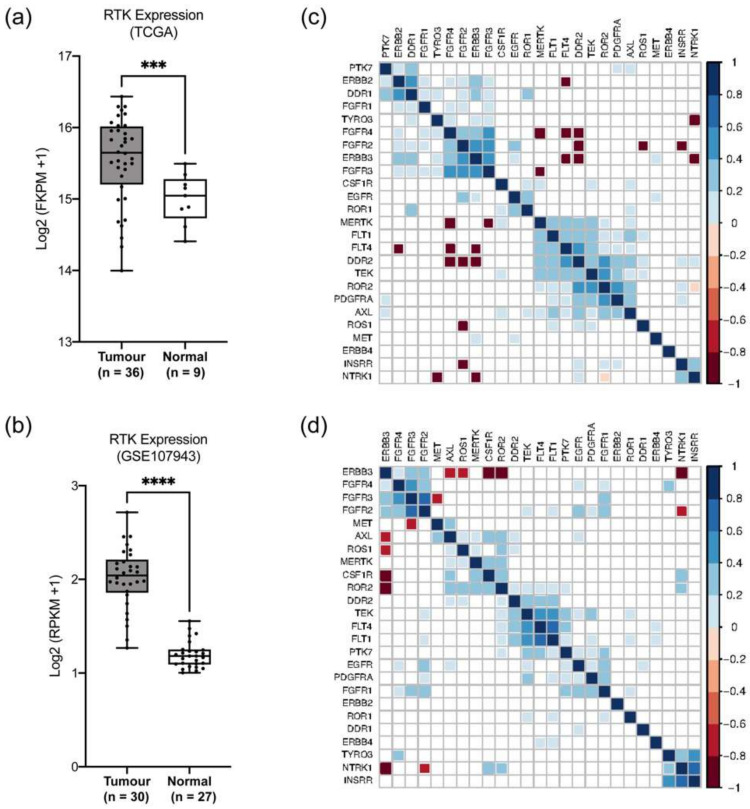
Validation of RTK25 in two independent RNA-seq databases. Boxplots represent the median value of averaged gene expression of RTK25 in CCA and normal tissues in (**a**) The Cancer Genome Atlas-Cholangiocarcinoma (TCGA-CHOL) and (**b**) GSE107943. ‘*n*’ refers to the number of samples in each group. The method for differential analysis between tumor and normal was one-way ANOVA testing followed by Holm–Sidak post-hoc analysis (*** *p* < 0.001, **** *p* < 0.0001). Correlation matrix represents significant Pearson correlation coefficients in (**c**) TCGA-CHOL and (**d**) GSE107943. Color indicates correlation (blue, positive correlation (*r* = 0 to 1); red, negative correlation (*r* = −1 to 0)). Area of the square indicates the value of corresponding Pearson’s correlation coefficients. FKPM: fragments per kilobase million; RPKM: reads per kilobase million.

Considering that our RTK25 gene set was able to segregate samples into distinct profiles of CCA, we wished to confirm this using independent cross-platform datasets to validate this model. For this purpose, we utilized RNA-seq data of cholangiocarcinoma tissues from TCGA-CHOL and a publicly deposited dataset GSE107943. We observed that the RTK25 gene signature was expressed in significantly higher levels in CCA tumors when compared with normal tissues (*p* < 0.05) (Figure 5a,b). In addition, testing these samples with the training set revealed that the samples from TCGA-CHOL predominantly exhibit RTK25 signature similar to that of cluster 4 (Figure S7) whereas the samples from the GSE107943 dataset are clustered with cluster 2 and 4 (Figure S8). Moreover, the correlation matrix with significant correlation coefficients (r) showed that the CCA tumors exhibited similar patterns of the RTK25 gene expression signature (Figure 5c,d). For example, samples with high EGFR subfamily expression also had high expression of the FGFR receptors and these RTKs were positively correlated in both datasets. Similarly, *AXL*, *MET*, and *ROS1* receptors were significantly positively correlated with each other in the GSE107943 dataset (Figure 5d). Furthermore, this molecular profiling strategy was able to sort RNA-seq data of biliary tract cancer cell lines from Cancer Cell Line Encyclopedia (CCLE), a publicly available database, into cluster 4 (Figure S9). Altogether, these results confirm that RTK25 is a potential molecular profiling strategy in CCA.

### 2.6. Several Tyrosine Kinase Inhibitors Were Enriched to Target the Different RTK Expression Profiles

**Figure 6:**
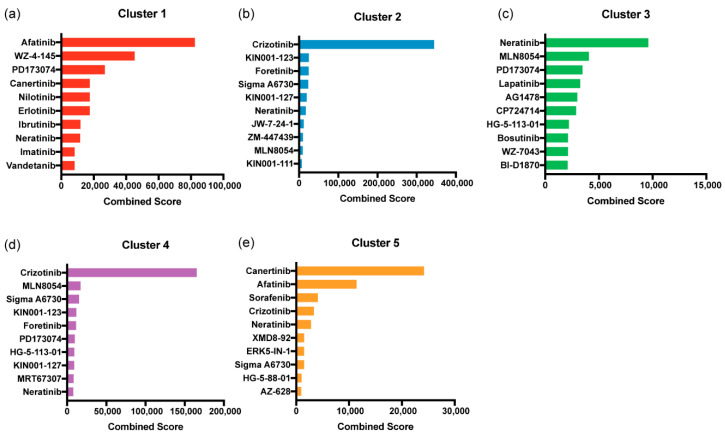
Enriched kinase inhibitors to target the RTK profile in each CCA cluster. The RTK profile of each CCA cluster was queried as gene list in Enrichr using the Harvard Medical School (HMS) KinomeScan Database. The resultant drugs that target the RTKs were filtered for significance (*p* < 0.05) and ranked according to the Enrichr combined score (also known as enrichment score) Reprinted from ref. [28]. The top 10 drugs enriched in (**a**) cluster 1, (**b**) cluster 2, (**c**) cluster 3, (**d**) cluster 4, and (**e**) cluster 5 are represented in the bar plot. Reprinted from ref. [18].

We surveyed the highly expressed RTKs in each of the clusters against the Harvard Medical School (HMS) KinomeScan database in Enrichr, to find small molecule kinase inhibitors that are significantly enriched for those RTKs. Additionally, we also sought to identify small molecule kinase inhibitors that are discordant or inversely enriched to the given differentially expressed gene signatures using iLINCS. Together, these results present enriched candidate molecules that target the distinct RTK profiles and can potentially reverse the molecular signature in each subtype. The resultant drugs that target the RTKs from HMS KinomeScan were retrieved and sorted according to the combined score and the top 10 drugs are reported (Figure 6). The RTK gene expression signature in cluster 1 resulted in enriched EGFR targeting inhibitors such as afatinib, canertinib, erlotinib, and neratinib. Moreover, multi-kinase inhibitors such as WZ-4-145, vandetanib, ibrutinib were also enriched for cluster 1. AXL, MERTK, and MET targeting kinase inhibitors such as crizotinib, foretinib, KIN001-127, neratinib, JW-7-24-1, ZM-447439, MLN8054, and KIN001-111 were significantly enriched for cluster 2. ERBB3 and FGFR3 targeting inhibitors such as neratinib, lapatinib, and PD173074 were significantly enriched for cluster 3. Some multikinase inhibitors such as AG1478, BI-D1870, HG-5-113-01, and MLN8054 were also enriched in Cluster 4, which had a mixed RTK signature. For the RTK gene signature in Cluster 5, mostly DDR1, ERBB4, and ROS1 inhibitors—such as sorafenib, afatinib, crizotinib, and canertinib—were enriched. Multi-kinase inhibitors that targeted DDR1, ERBB4, and ROS1 were also particularly enriched for this cluster.

Gene signature concordance from the iLINCS perturbation database was also evaluated using the DEG signature for each cluster when compared with the normal samples. We compared the DEG signature for each cluster with the signature of the drug perturbagens to identify which drugs can inhibit the upregulated genes for each cluster. We identified drug perturbagens from the ‘Cancer therapeutics response signatures’ database that reverses the input gene signature. Some of the drugs with discordant signatures were also observed in the enrichment analysis using Enrichr. The full list of significantly discordant drugs is provided in Supplemental Table S2. Erlotinib seemed to elicit the most discordant signature to the DEGs for cluster 1 and was also enriched to target the high-expressing RTKs in that cluster. This shows that the CCA samples in cluster 1 are most likely to respond to erlotinib. Similarly, RTK inhibitors such as cediranib, gefitinib, erlotinib, and crizotinib were discordant to the DEG signature for cluster 2. For cluster 3, JAK inhibitor, AZD-1480, and multi-targeted tyrosine kinase inhibitor, linifanib, showed a slight concordance. Likewise, afatinib, pazopanib, and crizotinib elicited the same DEG signature that was discordant to the DEG signature of cluster 4. Finally, cluster 5 was the most normal-like cluster; however, canertinib, afatinib, and neratinib showed concordance to its gene signature. Furthermore, sorafenib and crizotinib showed different levels of concordance in different cancer types, yet, seeing that these drugs were also enriched to target the RTK profile for cluster 5, they could be potential therapeutic candidates for the patients pertaining to this gene signature.

## 3. Discussion

CCA is a deadly disease with a dismal prognosis and a high mortality rate. Therefore, the increasing incidence of CCA is a cause for global concern. Treatment by surgical intervention is technically challenging; also, most patients are not well enough to withstand the toxicity of systemic therapy. Moreover, the recurrence rate, even after treatment, is high. Tumor heterogeneity, a prime characteristic of CCA, is a concern as it affects the response to treatment. Therefore, there is increasing evidence that supports the use of molecular profiling and biomarker-guided treatment in CCA. However, this effort is challenging due to the poor understanding of CCA’s genetic landscape. The advent of molecular profiling of CCA tumors has identified several molecular subtypes, including a specific subtype of patients harboring *FGFR2* fusions. This eventually led to the accelerated approval of Pemigatinib for patients with advanced metastatic CCA that harbor *FGFR2* fusions [9]. This expedited process of drug development highlights the urgent need for targeted therapy in CCA. Aberrant RTK signaling is a well-known phenomenon in CCA. Even though their potential as therapeutic targets has been explored to some extent, there is still a paucity of approved RTK targeted drugs in CCA. We believe that accurate patient stratification may improve the outcomes of clinical trials to launch more RTK targeted therapy in CCA management. While heterogeneous RTK expression is acknowledged in different cancer types, including CCA [29,30], there is still very little understanding of its molecular signature in cancer. This is the first meta-analysis study to report the comprehensive expression profile of RTKs in CCA at this scale.

Meta-analysis makes use of advanced statistical techniques to combine results from multiple independent studies. In this meta-analysis, we utilized publicly available microarray datasets of ten independent studies in CCA to identify a molecular profiling strategy. First and foremost, we have identified RTK genes with significance in CCA biology. We developed a molecular profiling strategy named RTK25, which discovered that there are five molecular subtypes in CCA. In addition, we also discerned candidate drugs that can target these molecular subtypes based on their gene signature. Firstly, we identified differentially expressed RTKs by comparing their expression in the tumors to that of the normal samples. We used the significantly dysregulated RTKs for the clustering analysis and for the optimal separation of clusters we used the RTK25 gene list. We discovered that, within this cohort, there are five subtypes with distinct RTK expression profiles. In addition to unsupervised clustering analysis, the multi-dimensional scaling analysis also verified the distinct transcriptomic differences between these clusters, hence classifying them uniquely. Therefore, the RTK25 molecular profiling strategy provides new insights into molecular characterization of CCA patients.

Crosstalks amongst the different RTKs are well-known in cancer. Besides, there are several reports of this phenomenon in other cancer types [31,32]. However, this area is not yet fully understood in CCA. Hence considering the tumor heterogeneity in CCA, we further evaluated the relationships amongst the RTKs using the Pearson correlation statistical test. Our findings show that the EGFR family and FGFR family are closely related. Also, AXL, MET, and MERTK are positively correlated while negatively correlated with other RTKs. These results suggest that there may be crosstalk between these RTKs in CCA. Furthermore, the RTK expression profile in the clusters corroborates with previous evidence that shows FGFR and EGFR working together in a manner of resistance against targeted therapies in other cancer types [33,34,35]. Ultimately, this compels future studies to investigate the efficacy and mechanism of dual-RTK inhibition as a therapeutic strategy in CCA.

Furthermore, we also identified inhibitors that can target the RTK profile of the different CCA clusters stratified using RTK25. We found that EGFR inhibitors (afatinib, erlotinib, and neratinib), FGFR inhibitor (PD173074), and AXL, MET inhibitor (crizotinib) were enriched to target the RTK profile in multiple clusters. This suggests that these drugs might be potential targeted therapy candidates for practicing precision medicine in CCA. However, more studies investigating the efficacy and understanding the underlying mechanism of these inhibitors are required in CCA. Previously, we reported that CCA cells expressing EGFR were sensitive to afatinib [36]. Here, we show that afatinib is enriched in clusters 1 and 5, which have high expression level of EGFR and ERBB2, and ERBB4, respectively. Altogether, this establishes proof of concept that biomarker-guided targeted therapy is feasible in CCA. Increasing evidence advocates the use of molecular profiling to identify subtypes of CCA and, to discover novel therapeutic strategies to target them [37]. Therefore, the RTK25 gene list can potentially be utilized for effective patient stratification and biomarker-guided therapy in CCA.

In addition, we also discovered several novel areas with knowledge gaps in CCA, in terms of the RTKs. We found that Eph receptors seemingly play a role in CCA biology; *EPHA1-4*, *EPHB3* were highly expressed whereas, *EPHA7*, *EPHA8*, *EPHA10*, *EPHB1*, *EPHB2*, *EPHB4*, and *EPHB6* had a lower expression in CCA tumors compared to normal tissues. However, we omitted these receptors from our RTK25 gene list for the optimal separation of the clusters. Nevertheless, the Eph receptors remain to be indispensable players in CCA pathogenesis and promising targets for treatment. Several reports illustrate the high Eph receptor expression and their potential as actionable targets in CCA [38,39,40]. Similarly, AXL receptor kinase is emerging as a promising target for anti-cancer therapy [41,42]. Our findings showed that *AXL* was expressed highly in CCA samples from clusters 2 and 4. Crizotinib is known to target and decrease AXL phosphorylation in other cancer types [43]. This is in line with our findings as crizotinib was one of the small molecules enriched in those clusters. Furthermore, data mining shows that CCA cell lines can be sorted into cluster 4 using RTK25 profiling (Figure S9) and are consequently sensitive to crizotinib (IC50 in μM: HuCCT-1, ~10.5; SNU1196, ~5.26, SNU1079, ~4.44; SNU869, ~6.85, SNU478, ~7.22) [44]. Moreover, we noticed that Pemigatinib, the only FDA approved targeted therapy drug in CCA, was not enriched for any of the clusters. One possible explanation for this is that Pemigatinib is a relatively new drug that was developed specifically for FGFR fusions in CCA, hence experimental data about the inhibitor is not yet publicly available. However, other FGFR inhibitors were enriched to target the CCA clusters with FGFR expression, which reiterates FGFRs as actionable targets in CCA. This further validates RTK25 molecular profiling strategy for therapeutic stratification. Altogether, the findings from this study can direct future investigative attention towards potential novel therapeutic strategies in CCA.

The main limitation of this meta-analysis is the lack of mutational profiling analysis of these tumors. The inclusion of mutational profiling would have provided more understanding of the underlying molecular mechanisms of CCA biology in the different clusters. As the datasets used in this meta-analysis were mainly from the microarray platform, the corresponding mutational data was not readily available for each study. However, this is the first report to describe the heterogeneous expression profile of RTKs in CCA. Moreover, the RTK25 strategy can also be utilized to characterize the molecular profile of CCA tissues using an RNA-sequencing platform as well, as illustrated in the validation analysis (Figures S7 and S8). In addition, the normal tissues used in this meta-analysis were collected from cancer-free tumor margins of CCA patients undergoing surgical resection. Hence, the normal tissues could also contain precursor cancerous cells. Therefore, future studies that investigate the RTK profile between CCA patients and healthy individuals may adequately address this limitation. In addition, the risk levels were not evaluated for the identified subtypes due to the lack of availability of the data; however, we believe this could be useful for predicting prognosis in CCA patients. Finally, this study also identified candidate drugs that can target the different molecular subtypes, however, further experimental validation of this strategy in preclinical in-vitro and in-vivo systems is warranted before it can be investigated in clinical trials.

In the long-term, our findings are expected to facilitate the incorporation of precision medicine techniques in clinical trials, in turn, this can expedite new drug development. As a result, novel therapeutics options for CCA management can be discovered. Altogether, we believe that this study has laid the foundation for future research involving RTKs in CCA. Therefore, RTK25 molecular profiling is expected to redefine patient stratification for personalized medicine in CCA.

## 4. Materials and Methods

### 4.1. Data Acquisition

Microarray gene expression data were obtained from Gene Expression Omnibus (GEO) using the search terms “cholangiocarcinoma” AND “human”. Results were filtered to select only tissue samples with baseline expression profiling. Ten datasets, containing a total of 704 CCA tumors, and 165 normal tissues were collated. Dataset information is summarized in Table 1.

### 4.2. Data Pre-Processing

Each dataset was normalized using quantile normalization. Subsequently, the datasets were annotated with corresponding gene IDs to probes in each respective platform. Upon collating the datasets and merging the gene IDs, the datasets were again normalized between arrays using quantile normalization. The gene expression data constructed using 10 different microarray datasets had a total of 704 tumors and 165 normal tissues samples. To this normalized expression data, we applied log2(x + 1) transformation. Processing was done using ‘R’ version 4.0.2. The schematic workflow of data acquisition and pre-processing is described in Figure 1.

### 4.3. Statistical Analysis

The normalized and transformed dataset was filtered using the RTK gene list (*n* = 54) obtained from HUGO Gene Nomenclature Committee (HGNC). Average expression was compared between CCA tumors versus normal tissues using one-way ANOVA followed by post-hoc analysis by Holm–Sidak’s multiple comparison test using GraphPad Prism 9. * *p* < 0.05, ** *p* < 0.01, *** *p* < 0.001, **** *p* < 0.0001. For correlation analysis, pairwise Pearson coefficient calculated for the selected RTKs and ordered matrix was generated using *corrplot* package, version 4.0.2 in ‘R’. Differential Gene Expression (DGE) analysis was performed using ‘limma’ package, version 4.0.2 in ‘R’.

### 4.4. Hierarchical Clustering

Our final gene list (RTK25) resulted in 25 RTK genes that were significantly differentially expressed in the CCA, this was used to conduct hierarchical clustering of the samples, using the ‘Complete’ clustering method and ‘Euclidean’ clustering distance. Heatmap was drawn using the *pheatmap* package, version 4.0.2 in ‘R’.

### 4.5. Gene Ontology and Pathway Enrichment Analysis

Gene Ontology (GO) and Pathway Enrichment analysis for the DEGs were obtained per cluster group when compared with normal using the web tool ‘Enrichr’ [18,35,36]. The resultant terms were filtered for statistical significance (*p* < 0.05) and ordered according to the combined score. The top 10 enriched results from GO cellular component, GO biological process, GO molecular function, BioPlanet, KEGG, MSigDB, and WikiPathways databases were represented.

### 4.6. Drug Enrichment Analysis

Enrichment of kinase perturbation for the RTK profile for each cluster was performed using Enrichr with utilizing KINOMEscan kinase inhibitor screening database [18,35,36]. Statistically significant kinase inhibitors (*p* < 0.05) were ordered according to combined score (also known as enrichment score [28]) and the top 10 results are reported. The DEG signature obtained for each cluster was compared with the gene signatures from chemical perturbation database in iLINCSs [45]. The concordance for the statistically significant perturbagens were evaluated.

### 4.7. Validation

We used FKPM (fragments per kilobase million) data from The Cancer Genome Atlas -Cholangiocarcinoma (TCGA-CHOL) which contains CCA (*n* = 36) and normal tissues (*n* = 9) and RPKM (reads per kilobase million) data from the GSE107943 dataset from GEO which also contains CCA (*n* = 30) and normal tissues (*n* = 27). The gene signature expression was conducted by averaging the expression of RTK25 in CCA and normal tissues using one-way ANOVA followed by post-hoc analysis by Holm–Sidak’s multiple comparison test using GraphPad Prism 9. * *p* < 0.05, ** *p* < 0.01, *** *p* < 0.001, **** *p* < 0.0001. Unsupervised clustering was performed using RTK25 gene list and pairwise correlation coefficient was calculated using Pearson method.

## 5. Conclusions

By using the RTK25 gene list, we identified that there are distinct RTK gene expression profiles in subsets of CCA samples. In some clusters, the EGFR and FGFR subfamily are highly expressed whereas in other clusters, *AXL, MET, MERTK,* and *TEK* are highly expressed. Understandably, EGFR and FGFR targeting inhibitors and multi-kinase inhibitors are enriched for the clusters with a high EGFR and FGFR expression profile. Similarly, multikinase inhibitors—such as crizotinib—were enriched for the subset of samples with high *AXL*, *MET*, and *MERTK* expression. However, there are also subsets of sample groups that have a mixed RTK signature outside of this pattern. Altogether, the results suggest that RTK expression is heterogeneous in CCA, hence it is important to explore their role in CCA further to identify novel therapeutic combinations for effective intervention. Ultimately, our findings will facilitate patient stratifications for treatment based on RTK expression with the potential to redefine precision medicine in CCA.

## Figures and Tables

**Table 1:** Databases used in the bioinformatics analysis of the gene expression profile in CCA.

GEO ID	Platform	CCA	Normal		Reference
			Bile Duct	Liver	
GSE132305	Affymetrix Human Genome U219 Array	182	38		[18]
GSE22633	Illumina Human-6 v2.0	20	4		[19]
GSE26566	Illumina HumanRef-8 v2.0	104	6	59	[20]
GSE32225	Illumina HumanRef-8 WG-DASL v3.0	149	6		[21]
GSE32879	Affymetrix Human Gene 1.0 ST Array	16	0	7	[22]
GSE35306	Affymetrix Human Gene 1.0 ST Array	3	0		[23]
GSE57555	Agilent-039494 SurePrint G3 Human GE v2	11	0	11	[24]
GSE66255	Illumina HumanHT-12 V4.0	10	17		[25]
GSE76297	Affymetrix Human Transcriptome Array 2.0	91	92		[26]
GSE89749	Illumina HumanHT-12 V4.0	118	2		[27]
Total		704	165		

## Data Availability

Data is contained in article and Supplementary Materials.

## Supplementary Materials

The following are available online at https://www.mdpi.com/article/10.3390/ph14090898/s1, Figure S1: Differentially Expressed Genes (DEGs) in each cluster group; Figure S2: Enriched Pathways for Cluster 1; Figure S3: Enriched Pathways for Cluster 2; Figure S4: Enriched Pathways for Cluster 3; Figure S5: Enriched Pathways for Cluster 4; Figure S6: Enriched Pathways for Cluster 5; Figure S7: Validation of the molecular profiling using cholangiocarcinoma samples from The Cancer Genome Atlas (TCGA-CHOL) database; Figure S8: Validation of the molecular profiling using cholangiocarcinoma samples from publicly available dataset (GSE107943); Figure S9: Validation of the molecular profiling using cholangiocarcinoma cell lines from publicly available data in Cancer Cell Lines Encyclopedia (CCLE); Table S1: The RTK gene list used in this study; Table S2: Discordant signature match with DEGs from iLINCs.
