# Supplementary material for: RTK25: A Comprehensive Molecular Profiling Strategy in Cholangiocarcinoma Using an Integrated Bioinformatics Approach
Source: Pharmaceuticals (Basel). 2021 Sep 3;14(9):898. doi: 10.3390/ph14090898 (PMC8469883; doi:10.3390/ph14090898)
Supplement: Supplementary file 1 [file pharmaceuticals-14-00898-s001.zip › Supplementary File 1.pdf]

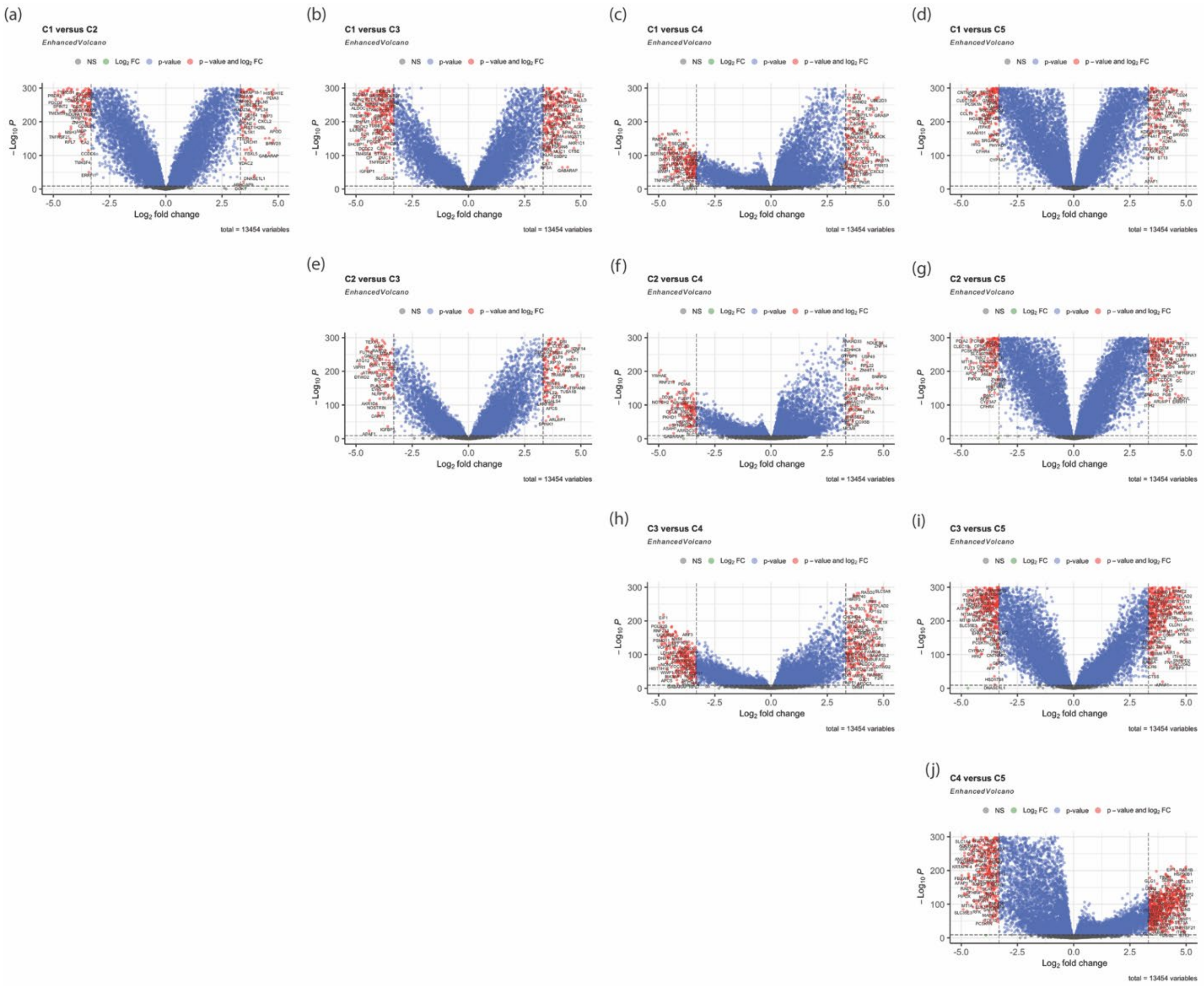


**Figure S1**. Differentially expressed genes (DEGs) in each cluster group. DEGs when comparing cluster 1 against cluster (a) 2, (b) 3, (c) 4, (d) 5; cluster 2 against cluster (e) 3, (f) 4, (g) 5; cluster 3 against cluster (h) 4, (i) 5; cluster 4 against cluster (j) 5. Statistically significant DEGs which meet the fold change cut-off are represented by red dots (adjusted $p < 0.001$), blue dots represent significant DEGs beyond the log2 fold change cut-off (adjust-ed $p < 0.001$), and grey dots represent non-significant genes. C1: cluster 1; C2: cluster 2; C3: cluster 3; C4: cluster 4; C5: cluster 5.

(A)

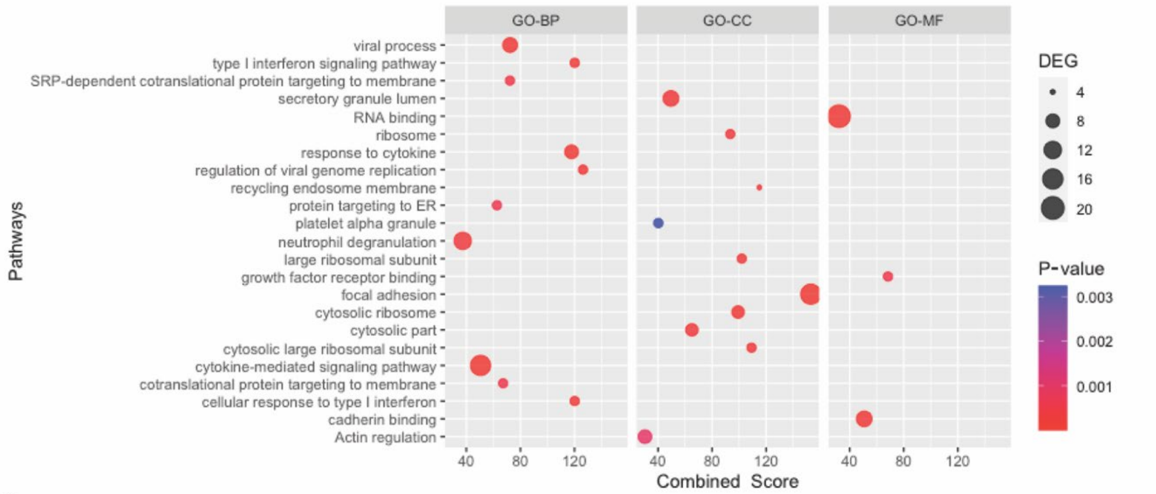


(B)

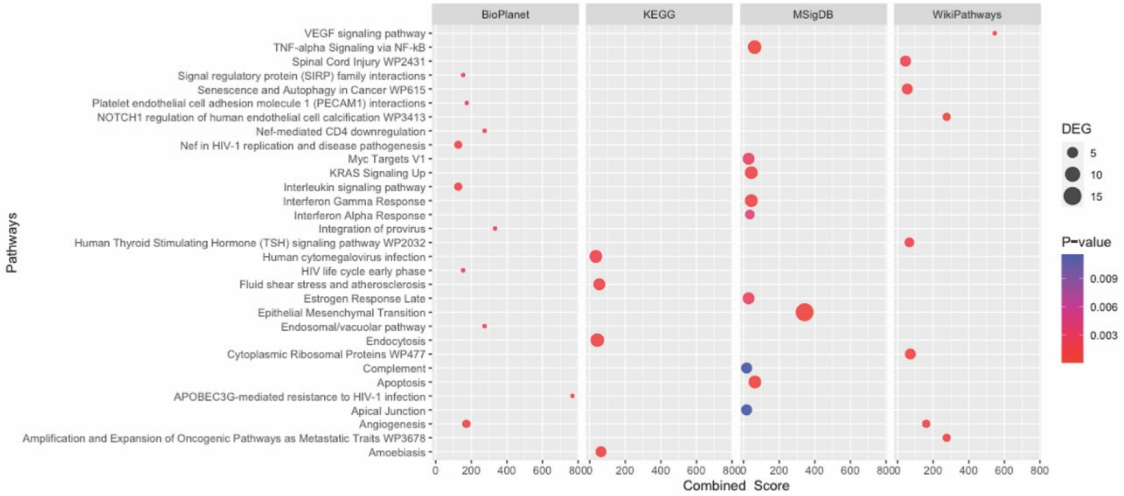


**Figure S2** Enriched Pathways for Cluster 1. The unique DEGs for cluster 1 were used to identify enriched pathways from the Gene Ontology (GO-BP: Biological Process; GO-CC: Cellular Component; GO-MF: Molecular Function), BioPlanet, Kyoto Encyclopaedia of Genes and Genomes (KEGG), The Molecular Signatures Database (MSigDB) and WikiPathways. Combined score (also known as enrichment score) is a score for ranking the enriched terms, the higher the score the more relevant they are with the DEGs. The size of the bubble node stands for the number of DEGs per enriched pathway. The colour of the bubble node represents the p-value. Bubble plot was constructed using the *ggplot2* package in R.

(A)

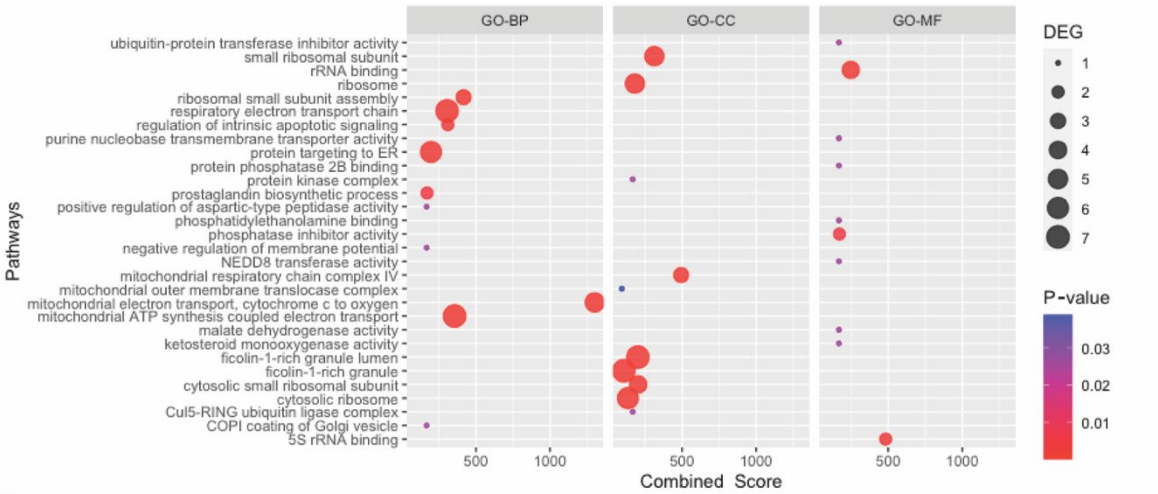


(B)

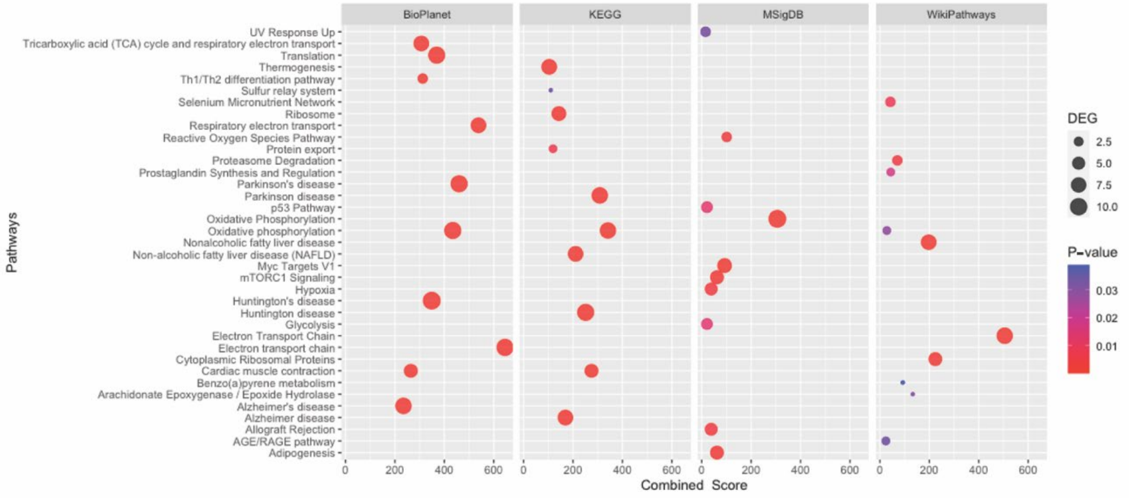


**Figure S3** Enriched Pathways for Cluster 2. The unique DEGs for cluster 2 were used to identify enriched pathways from the Gene Ontology (GO-BP: Biological Process; GO-CC: Cellular Component; GO-MF: Molecular Function), BioPlanet, Kyoto Encyclopaedia of Genes and Genomes (KEGG), The Molecular Signatures Database (MSigDB) and WikiPathways. Combined score (also known as enrichment score) is a score for ranking the enriched terms, the higher the score the more relevant they are with the DEGs. The size of the bubble node stands for the number of DEGs per enriched pathway. The colour of the bubble node represents the p-value. Bubble plot was constructed using the *ggplot2* package in R.

(A)

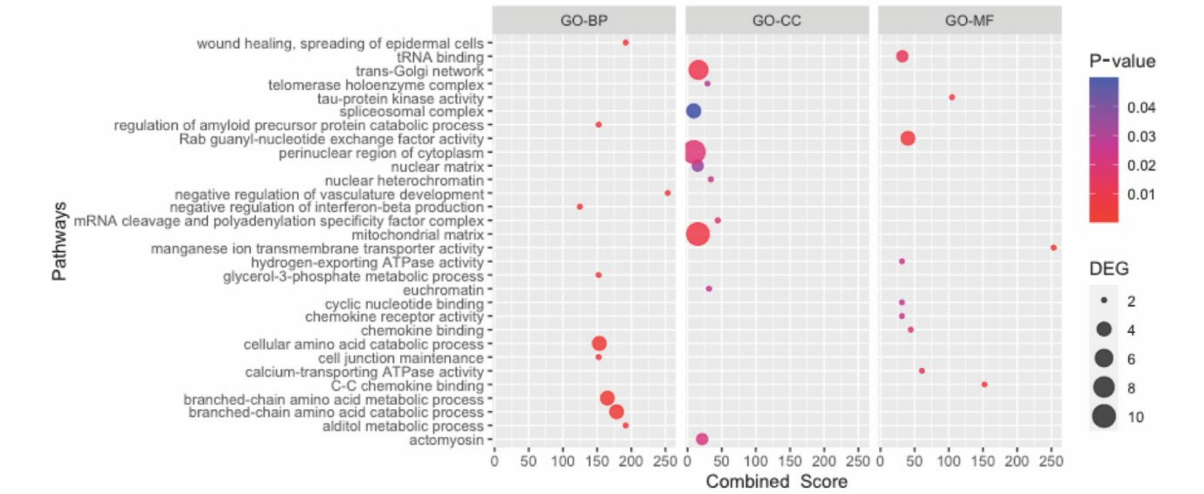


(B)

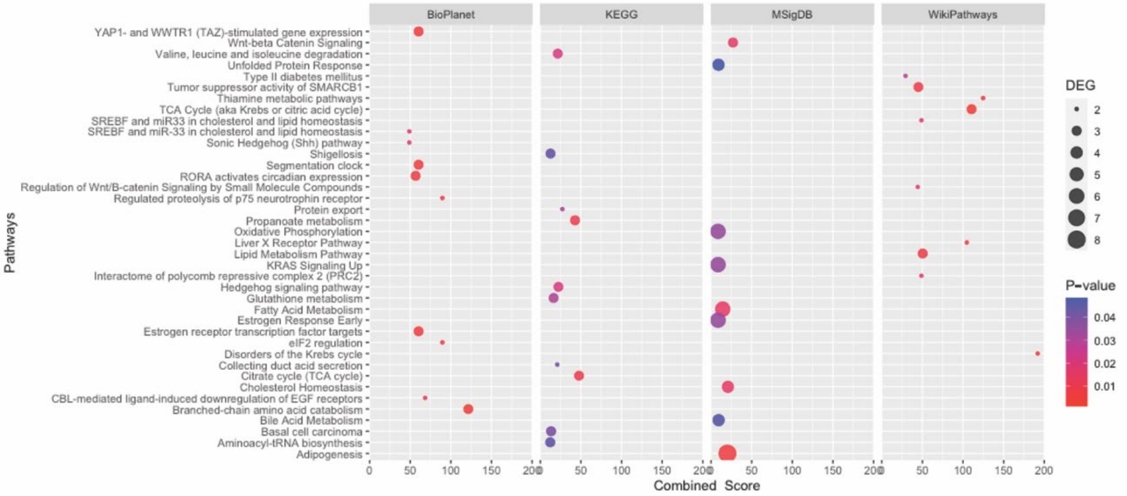


**Figure S4** Enriched Pathways for Cluster 3. The unique DEGs for cluster 3 were used to identify enriched pathways from the Gene Ontology (GO-BP: Biological Process; GO-CC: Cellular Component; GO-MF: Molecular Function), BioPlanet, Kyoto Encyclopaedia of Genes and Genomes (KEGG), The Molecular Signatures Database (MSigDB) and WikiPathways. Combined score (also known as enrichment score) is a score for ranking the enriched terms, the higher the score the more relevant they are with the DEGs. The size of the bubble node stands for the number of DEGs per enriched pathway. The colour of the bubble node represents the p-value. Bubble plot was constructed using the *ggplot2* package in R.

(A)

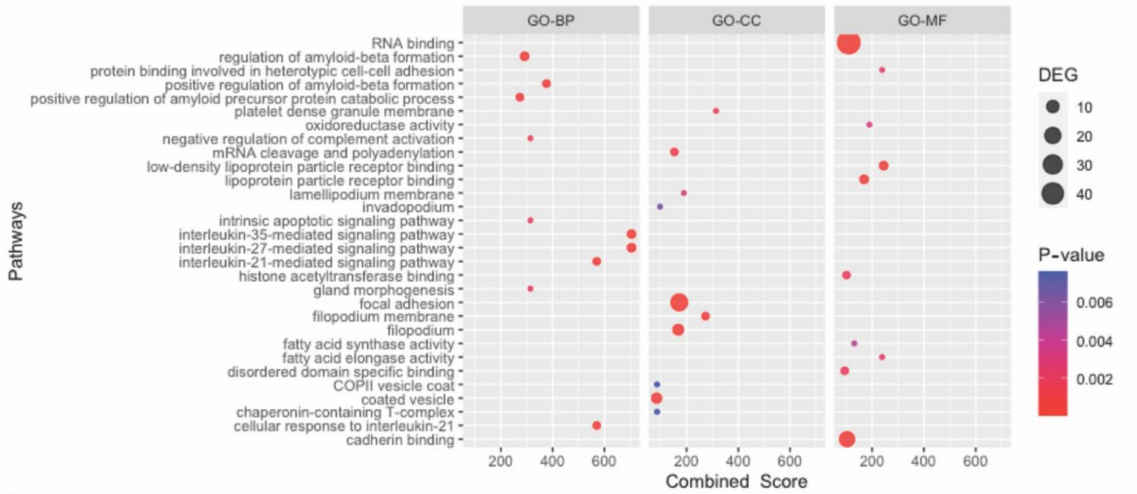


(B)

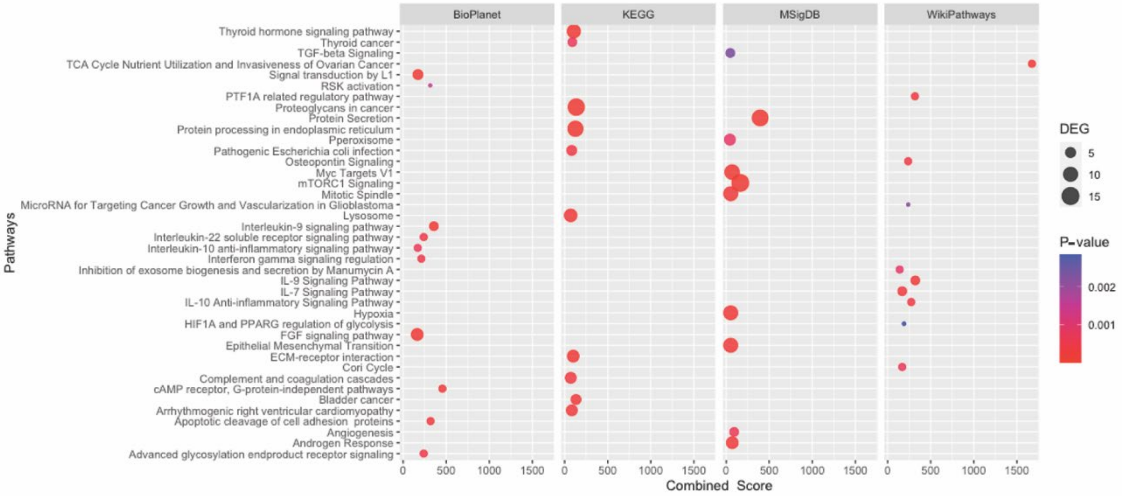


**Figure S5** Enriched Pathways for Cluster 4. The unique DEGs for cluster 4 were used to identify enriched pathways from the Gene Ontology (GO-BP: Biological Process; GO-CC: Cellular Component; GO-MF: Molecular Function), BioPlanet, Kyoto Encyclopaedia of Genes and Genomes (KEGG), The Molecular Signatures Database (MSigDB) and WikiPathways. Combined score (also known as enrichment score) is a score for ranking the enriched terms, the higher the score the more relevant they are with the DEGs. The size of the bubble node stands for the number of DEGs per enriched pathway. The colour of the bubble node represents the p-value. Bubble plot was constructed using the *ggplot2* package in R.

(A)

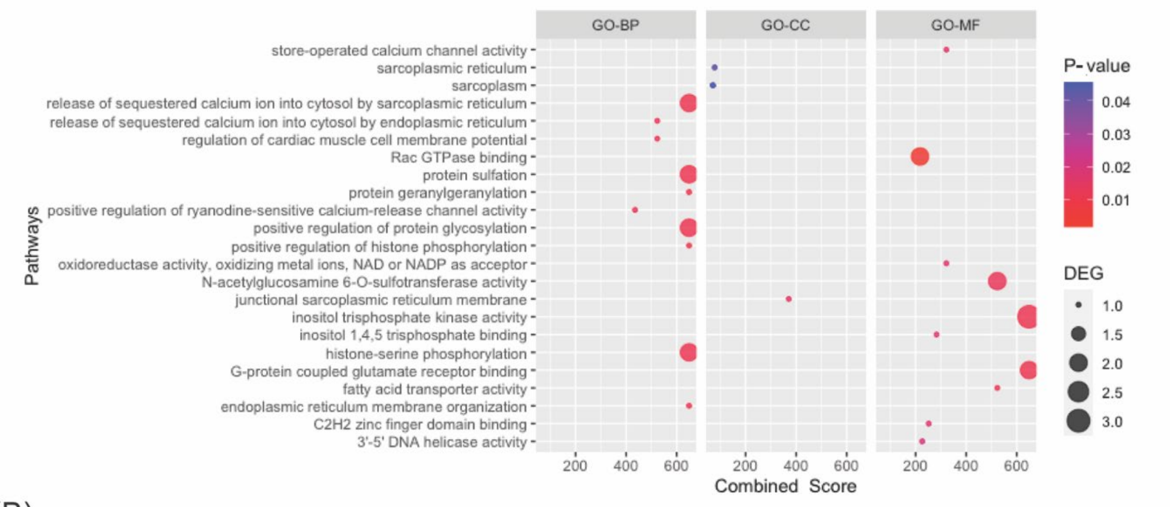


(B)

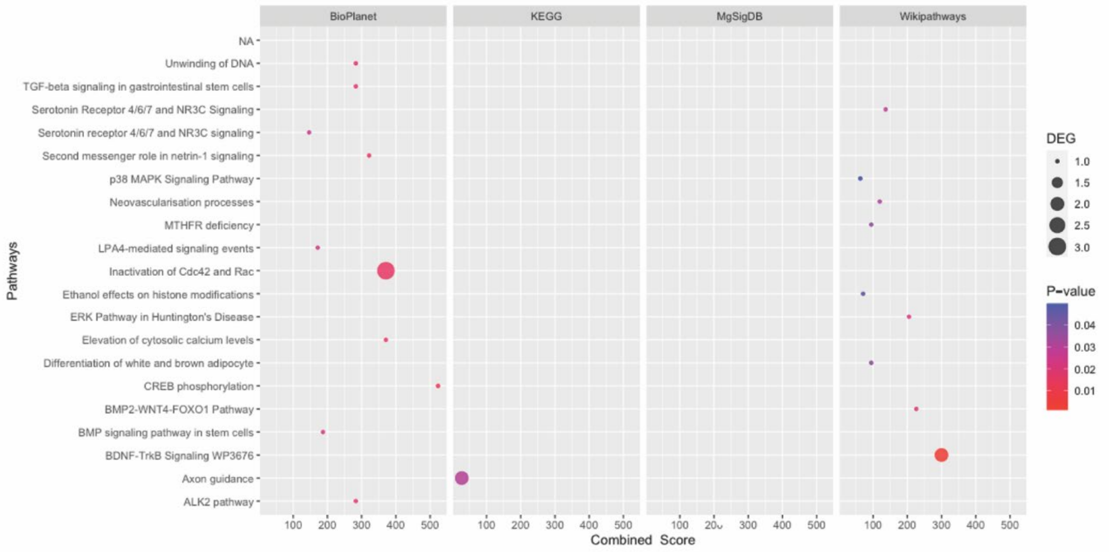


**Figure S6** Enriched Pathways for Cluster 5. The unique DEGs for cluster 5 were used to identify enriched pathways from the Gene Ontology (GO-BP: Biological Process; GO-CC: Cellular Component; GO-MF: Molecular Function), BioPlanet, Kyoto Encyclopaedia of Genes and Genomes (KEGG), The Molecular Signatures Database (MSigDB) and WikiPathways. Combined score (also known as enrichment score) is a score for ranking the enriched terms, the higher the score the more relevant they are with the DEGs. The size of the bubble node stands for the number of DEGs per enriched pathway. The colour of the bubble node represents the p-value. Bubble plot was constructed using the *ggplot2* package in R.

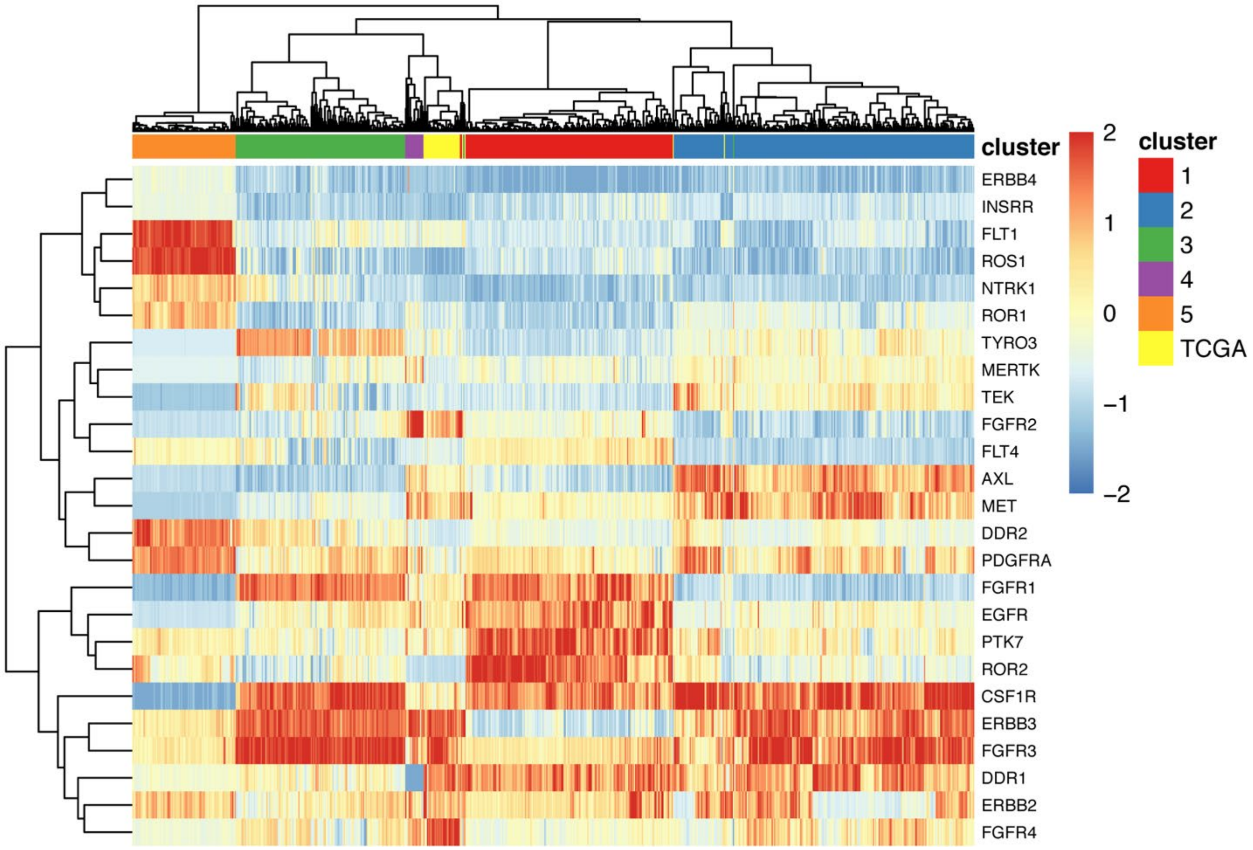


**Figure S7.** Validation of the molecular profiling using cholangiocarcinoma samples from The Cancer Genome Atlas (TCGA-CHOL) database. Hierarchical clustering was performed on the training set (n = 704) with the validation test (n = 36) based on the gene expression of selected RTKs (RTK25). The heatmap indicates the expression values (red indicates high expression and blue indicates low expression) of RTK25 in each cluster (red = 1, blue = 2, green = 3, purple = 4 and orange = 5, and yellow = TCGA-CHOL).

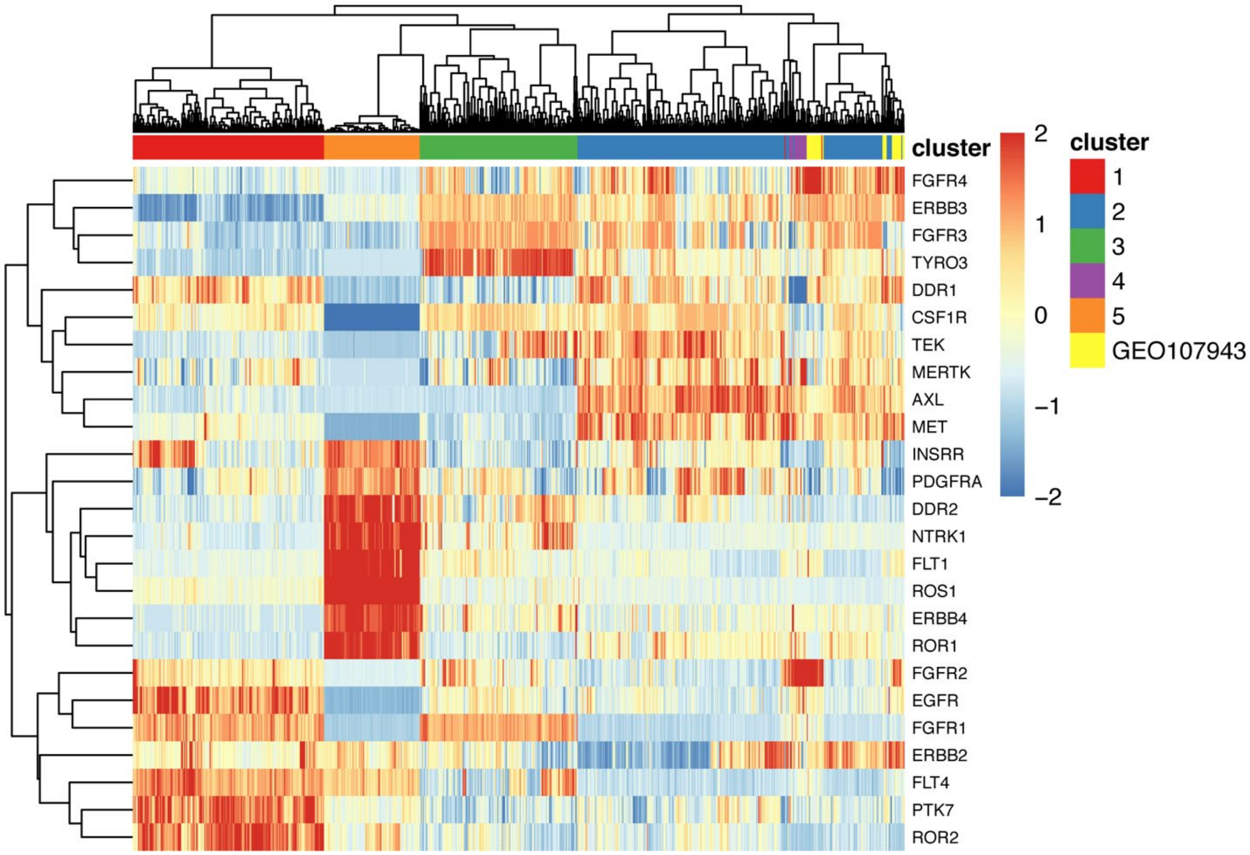


**Figure S8.** Validation of the molecular profiling using cholangiocarcinoma samples from publicly available dataset (GSE107943). Hierarchical clustering was performed on the training set (n = 704) with the validation test (n = 30) based on the gene expression of selected RTKs (RTK25). The heatmap indicates the expression values (red indicates high expression and blue indicates low expression) of RTK25 in each cluster (red = 1, blue = 2, green = 3, purple = 4 and orange = 5, and yellow = GSE107943).

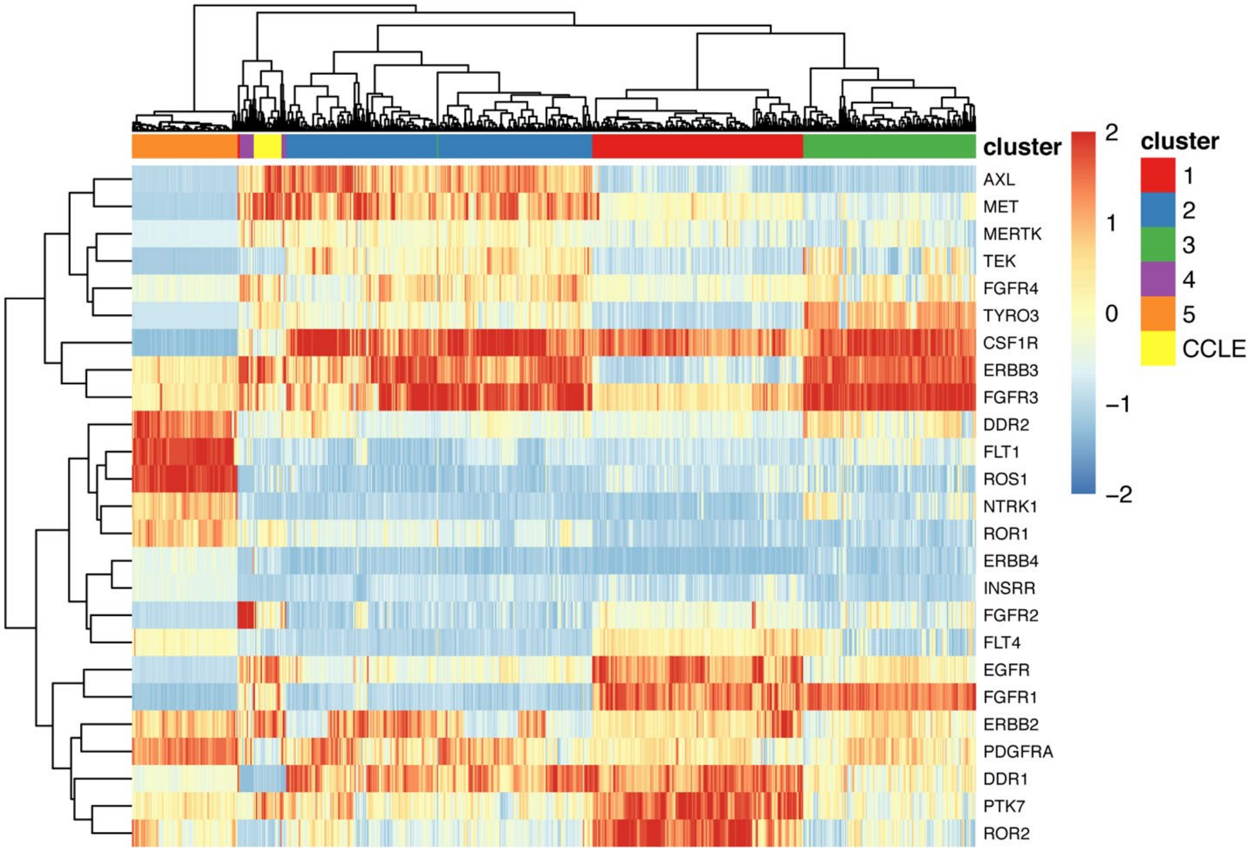


**Figure S9.** Validation of the molecular profiling using cholangiocarcinoma cell lines from publicly available data in Cancer Cell Lines Encyclopedia (CCLE). Hierarchical clustering was performed on the validation set (n = 704) with the CCLE set (n = 24) based on the gene expression of selected RTKs (RTK25). The heatmap indicates the expression values (red indicates high expression and blue indicates low expression) of RTK25 in each cluster (red = 1, blue = 2, green = 3, purple = 4 and orange = 5, and yellow = CCLE).

**Table S1.** The RTK gene list used in this study (RTK25).

| RTK Class | RTK Family | Genes |
|---|---|---|
| 1 | EGF | EGFR, ERBB2, ERBB3, ERBB4 |
| 2 | Insulin | INSRR |
| 3 | PDGF | PDGFRA, CSF1R |
| 4 | VEGF | FLT1, FTL4 |
| 5 | FGF | FGFR1, FGFR2, FGFR3, FGFR4 |
| 6 | CCK | PTK7 |
| 7 | NGF | NTRK1 |
| 8 | HGF | MET |
| 10 | AXL | AXL, TYRO3, MERTK |
| 12 | RYK | TEK |
| 13 | DDR | DDR1, DDR2 |
| 15 | ROS | ROS1 |
| 17 | ROR | ROR1, ROR2 |
